# Conservation futures 2050: Developing future scenarios to explore potential socio-economic developments and their impact on biodiversity

**DOI:** 10.1371/journal.pone.0311361

**Published:** 2024-12-12

**Authors:** Jennifer L. Bufford, Angela J. Brandt, Anne-Gaelle Ausseil, Amanda Black, Bradley S. Case, Adam Sean Forbes, Catherine Kirby, Rowan Sprague, Anita Wreford, Duane A. Peltzer

**Affiliations:** 1 Manaaki Whenua–Landcare Research, Lincoln, New Zealand; 2 Manaaki Whenua–Landcare Research, Dunedin, New Zealand; 3 Ministry for the Environment, Wellington, New Zealand; 4 Bioprotection Aotearoa, Lincoln University, Lincoln, New Zealand; 5 School of Science, Auckland University of Technology, Auckland, New Zealand; 6 Forbes Ecology Limited, Hastings, New Zealand; 7 Entelea Scicom, Hamilton, New Zealand; 8 Faculty of Environment, Society and Design, Lincoln University, Lincoln, New Zealand; Michigan State University, UNITED STATES OF AMERICA

## Abstract

Large scale changes in biodiversity and conservation management require long-term goals and planning across multiple sectors in the face of increasing global change. Major trends in land use and management interventions, species additions or losses, and climate are well recognized, but responses are still often short-term and fragmented across agencies and sectors. Scenario-building can be a powerful tool to imagine possible futures, integrating across sectors and disciplines and promoting long-term thinking and planning. As an interdisciplinary team of experts, we developed potential scenarios for a range of future environmental conditions. The scenarios explored: increasing land ownership and stewardship of land by indigenous peoples (Māori); widespread afforestation using native tree species; national-scale eradication of invasive mammalian predators; and increasing frequency of extreme weather events. We explored the implications of these globally-relevant trends at a national scale using Aotearoa New Zealand as our study system. Detailed descriptions of these scenarios were developed by experts using environmental, economic, social science and policy lenses. Across scenarios several common themes were consistently highlighted, including the importance of land use in driving other conservation outcomes. How the value of ecosystem services is recognized and prioritized was also important to a wide range of outcomes. Furthermore, each scenario presented both opportunities and risks to equality, indigenous empowerment and human capital, emphasizing the importance of good policy responses to maximize benefits and minimize unintended harm. These scenarios will be used to stimulate new questions and ideas for biodiversity conservation and management, such as considering the implications of different potential futures for the management of biological invasions. This approach is explicitly designed to be generalisable across different sites or regions and provides a method for considering the implications of potential future changes for a broad range of disciplines or needs.

## Introduction

In the face of the growing number, complexity and uncertainty of environmental challenges, long-term planning is essential for successful conservation programmes ranging from native species recovery to management of non-native invasive species [1–3]. Effectively linking research, management and policy planning using long-term strategic approaches is required to meet the challenges of uncertain future dynamics in potentially non-analogue future climates and ecosystems [4–7]. However attempts are often limited by relatively short-term funding and decision-making cycles coupled with known lags between science and policy development [8–11]. Exploring potential futures can direct research programmes towards new areas of need and can help practitioners prepare for, and respond proactively to, emerging challenges and make decisions robust to existing uncertainties [12–14]. Strategic foresight, therefore, has been advocated as a powerful tool for future-focused decision-making in the face of uncertainties in conservation, but is yet to be widely applied [12].

Scenario development is a form of strategic foresight that considers possible futures from multiple angles. Scenarios can explore a broad range of potential implications of an event or trend within a given timeframe, including indirect and complex effects [15–17]. This provides an approach for evaluating what futures we choose to work towards and revealing potential challenges and opportunities that could emerge based on current trends [17]. Scenario-building is also a mechanism for integrating different expertise and knowledge across disciplines and stakeholders, thus generating trends and perspectives that are more broadly informed and less constrained by individual lines of investigation or practice [15]. Scenarios therefore are powerful as a means of stimulating long-term thinking and planning beyond normal planning horizons in conservation research and practice [12, 15]. Planning for non-analogue futures, for example through future scenarios, is urgently required given current impacts and rapid changes in multiple drivers of global change [18, 19]. For example, the costs and damage from non-native invasive species are already a major global issue that could be exacerbated by climate change or increased weather extremes [20].

Aotearoa New Zealand (AoNZ) is an excellent study system for exploring these environmental and conservation issues, as island nations are often on the forefront by necessity because of their unique biota (i.e., high rates of endemicity), smaller geographic extent for range adjustment of species and relatively high rates of invasion by non-native species [21, 22]. Conservation in AoNZ is characterized by an emphasis on protecting rare and endangered species, particularly birds, and managing large tracts of remote, uninhabited native forest [2]. The lack of any native terrestrial mammals, except for native bats, has made the flora and fauna highly vulnerable to non-native mammalian predators and herbivores, some of which also have agricultural impacts. Much of the conservation work is focused on controlling these species [23], as well as high-impact invasive plants such as conifers [24] and an increasing number of emerging species [25]. Primary production is also vulnerable to non-native species and environmental changes [26] and provides key exports from AoNZ, including dairy, sheep and beef, forestry using non-native tree species, and fruit products (e.g. kiwifruit, apples, wine) [27]. Tourism is also a major component of the economy, long marketed under a “clean, green New Zealand” campaign. Given the wide-ranging potential impacts of these drivers of environmental change, there is interest in long-term, coordinated and transdisciplinary approaches to research, management and planning, but the scope of the challenges means many responses are still reactionary, isolated and occur within narrow boundaries.

As a result of this biogeographical and socio-economic context, AoNZ has begun addressing environmental issues including climate change and invasive species, and related social issues including land rights and conflicting land uses, which have often been neglected elsewhere [26]. For example, AoNZ has developed world-leading expertise in predator control and has some of the strictest biosecurity regulations in the world, producing a model that has been considered globally [28]. Natural disasters and weather extremes have already prompted forced relocations of residents in badly affected areas, with government buy-outs of unliveable homes and limits on rebuilding following natural disasters. This trend mirrors conversations underway in many other countries [19]. Indigenous Māori groups have campaigned for decades for better recognition of their rights and redress of historical harms (e.g. illegal land seizure) under AoNZ’s founding treaty, the Treaty of Waitangi–Te Tiriti o Waitangi, resulting in increasing formal recognition of Māori language and culture and the return of land to a form of indigenous governance or co-governance [29–32]. Similar underlying issues exist globally, including in Canada, the United States, India and Brazil, although the extent to which they have been or are being addressed varies widely [33–35].

We have therefore developed a set of possible future scenarios that span a range of drivers that reflect trends operative globally and explored their implications for AoNZ. However, these scenarios can aid managers or those conducting similar exercises across a range of settings. Here we describe our approach to developing the scenarios, highlight the strengths and limitations of this approach, and describe ways in which these scenarios can be used to stimulate conservation thinking and planning over a longer time horizon than is usually considered [8].

## Materials and methods

The goal of this exercise was to develop scenarios that drew on and extended current trends and ideas to imagine a suite of non-exclusive possible futures for AoNZ in 2050, exploring their potential environmental, policy, economic and social implications [12, 15]. We chose 2050 because we expect a 30-year timeframe to provide time for significant change to begin, it is about one human generation which is a timescale often considered in scenarios, and it is sufficiently proximate that current decisions or management activities can influence the outcome in traceable ways. A 30-year timeframe is also at the far end of most research and management planning horizons but is still within the timeframes considered for some very long-term planning, such as the Predator Free 2050 programme, which aims to eradicate three groups of non-native mammalian predators from AoNZ by 2050 [36].

We developed scenarios that confronted current research and management paradigms with significant change but were plausible and could prompt proactive research and policy in the near-term. Unlike many scenario exercises, which focus on a single core issue and construct scenarios that represent a range of potential responses [17], we chose to address a non-exclusive range of potential issues. We wanted to generate scenarios that drew from the trends, priorities and concerns considered by different disciplines and spanned multiple long-term drivers of environmental change. This approach also reflects conservation and research planning needs that are developed in the context of multiple non-exclusive potential threats, issues and opportunities [2]. We therefore used a modified scenario-building structure, which selected several core developments and briefly explored the pathways, opportunities and risks within each one, allowing us to cover more developments with the time available. How a scenario is achieved is often just as important as the scenario itself, so we highlighted the key uncertainties and contingencies that can shape outcomes, including research agendas, management priorities and policy (detailed below).

**Fig 1:**
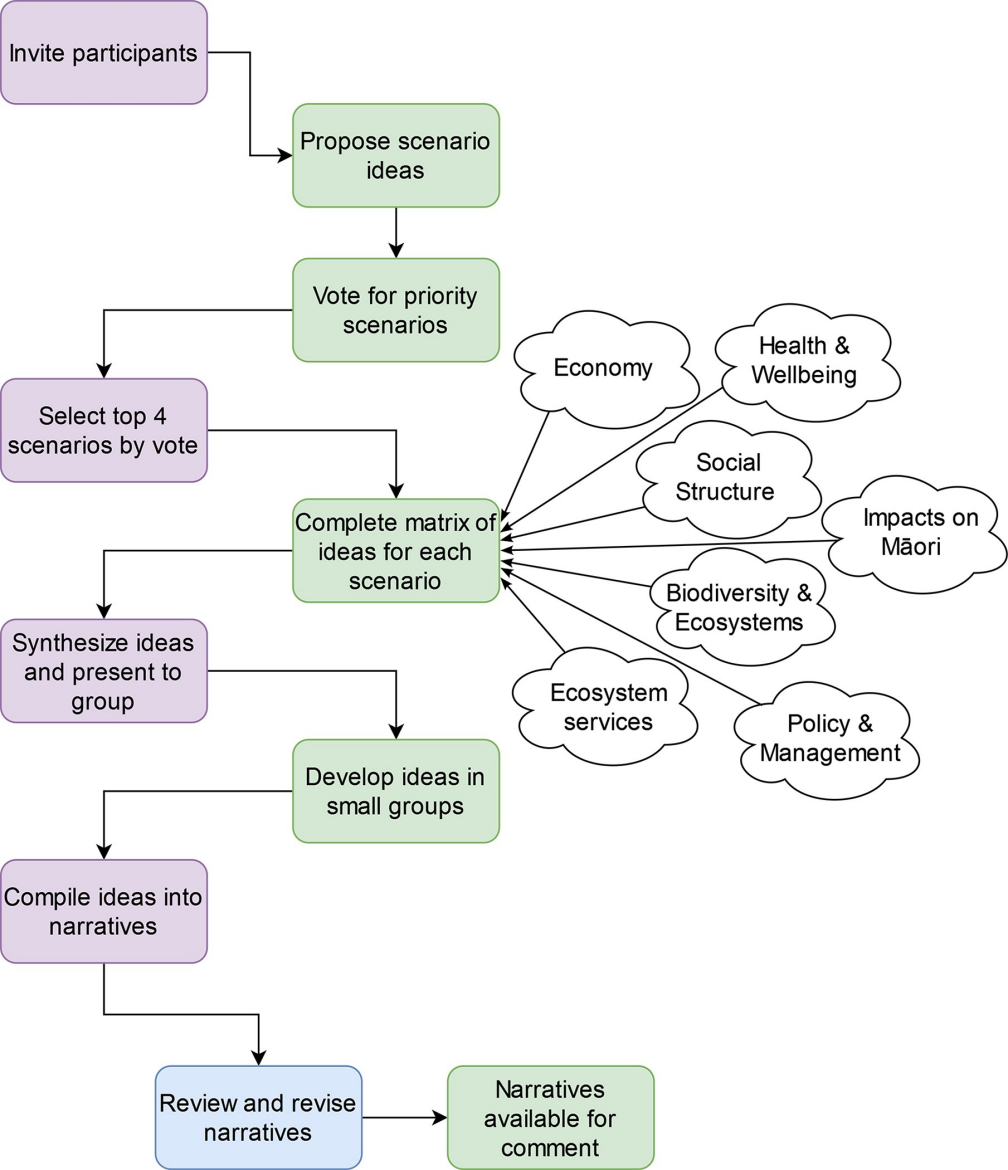
The process of developing the scenario narratives is illustrated, with purple boxes showing actions taken by the core authors (JLB, AJB, DAP) and the green boxes showing activities done with the full set of workshop participants. The blue box shows action taken by the authors of this manuscript. White clouds show the dimensions that workshop participants considered when developing the scenarios. The scenario ideas initially proposed, and the questions used to stimulate thinking about the scenarios are available at [37].

We solicited scenario ideas from 15 participants with expertise spanning conservation and biosecurity management and policy, invasive predator control, climate change, economics, social science, forestry, indigenous rights and environmental monitoring (Fig 1). Participants came from universities, research organisations, central government agencies, local government and consultancies, and included both established and emerging researchers, people originally from AoNZ and from overseas, and indigenous peoples. Because scenario development is always subjective and influenced by the areas of expertise of those involved, we invited a diversity of participants to capture developments in a range of fields [15]. Due to the time commitment involved, we were not able to engage any current land managers or practitioners directly, but many participants had experience working directly with practitioners. To encourage participation from people across this range of backgrounds and career stages, many key processes in the scenario development were either completely anonymous, or individual identity was only visible to the organizers (JLB and AJB), and other stages of the process focused on working in small groups. We asked each participant to propose at least one potential future scenario for 2050 based on their knowledge and trends in their field of expertise. We then collated the proposed scenario descriptions to reconcile similar ideas, with a final list of 10 potential scenarios for consideration (available at [37]). To feasibly explore the scenarios in detail, we selected a subset of contrasting scenarios for further development. The participants voted anonymously for their top three choices and explained their reasoning. Four scenarios clearly had more support than the others, and often could incorporate ideas from the other six proposals, so these four were selected for further development. Our chosen scenarios focused on: Māori land tenure, native afforestation, predator eradication and extreme climate events (Table 1). These four scenarios together covered a range of possible dynamics, included major national trends the group considered highly relevant, and were plausible but required a stretch in thinking from business as usual.

**Table 1:** Brief description of four future scenarios for Aotearoa New Zealand in 2050. Each scenario was considered and developed in detail. Full scenario description and potential implications are available online [37].

Scenario Name	Brief Description
**Māori Land Tenure**	As a result of Treaty settlements and changes in governance structure, indigenous Māori communities manage large tracts of land and Māori are consistently represented in land management decision-making locally and nationally.
**Native Afforestation**	Native production forest replaces the use of non-native (exotic) trees for commercial harvest and native permanent forest is created through reforestation of marginal lands for ecosystem services including carbon sequestration.
**Predator Eradication**	Rats, brushtail possums and mustelids are heavily suppressed or functionally eliminated nationwide as a result of a largely successful Predator Free 2050 programme, with some increase in control of other invasive mammals.
**Increasing Extreme Events**	Climate change results in increasing frequency, scale and intensity of extreme events, including fires, storms and flooding, with significant impacts on infrastructure, funding policies and persistence of native biodiversity.

We convened a one-day workshop with the participants to discuss and develop the selected scenarios (Fig 1). Each participant was given a matrix beforehand which asked them to consider policy and management, economic, social and environmental implications for each scenario, including new drivers and trends, exacerbated dynamics and indirect or cascading effects (available at [37]). Participants were encouraged to discuss the scenarios with colleagues and gather opinions and ideas from others. We compiled, anonymized and summarized feedback before the workshop and then discussed these ideas further in person, challenging and consolidating our ideas and building a picture of the way the changes we identified would most likely affect society and environmental management. We used a scenario workshop format to engage and stimulate creativity and build collaborations by splitting into small groups for brainstorming, using semi-structured questions, open sketches and diagrams, and free-form conversation.

From the ideas and issues discussed and text developed in the workshop, we then generated a detailed written narrative, and accompanying summary, for each scenario. This was done by synthesizing the key points raised in the contributions both before and during the workshop, with an emphasis on those points that participants felt were most important, or that were echoed by multiple participants. We also considered which points could be supported by examples from the literature or from the experience of the participants. Each scenario is built around a specific trend or future development which represents a significant departure from the *status quo*, and the potential opportunities and risks for environmental, policy, economic and social factors were identified. We discuss the pathways leading to each scenario and explore the impacts of the scenario, including uncertainties and a range of possible outcomes.

## Results

We developed four different scenarios in detail. Within each scenario, we discussed the possible pathway to 2050 and the range of direct and indirect effects, uncertainties, counter-intuitive effects and context-dependencies. Summary narratives for each scenario are presented below. These provide a brief description of the most important and most likely effects, while the full scenario descriptions explore the range of more complex possible outcomes. For those interested in using these scenarios in their own planning exercises, the detailed descriptions are available online [37].

### Māori land tenure

Increased Māori land tenure and representation can come through Treaty settlements, trust or corporation-owned land including through private sales, or consultation and policy-driven co-governance over Crown-held land. Māori land is currently legally classified by use and management, with separate categories for land managed in accordance with tikanga (Māori customs), freehold land and land designated for specific uses, such as for historical or spiritual reasons [38]. These mechanisms represent a range of autonomy in decision-making, access to funding, and condition and possible use of land [30, 31, 39]. Under this scenario, environmental opportunities include the use of indigenous knowledge to renew cultural practices and improve ecological connectivity between people and environment (i.e. biocultural renewal) [35, 40]. Key environmental risks include constraints caused by lack of funding or the need for income generation and the effects of climate change, as well as the disruptive effects of changes in management regimes [39, 41].

Increased biculturalism and decentralization can provide policy opportunities, but existing systems and capacity limits may prevent the realisation of Māori aspirations, exacerbate inequalities and limit coordination [30, 42]. Economic shifts will depend on the extent to which land use changes with increased Māori control or influence and the extent to which current systemic barriers are removed. Opportunities could include diversification and new products based on native species and cultural traditions with an increased emphasis on profits returning to local communities [41, 43]. However, the uncertainties and lag time between developing new land uses and seeing economic benefits, as well as increased demand on Māori time and resources, pose risks for economic loss and may limit the extent of any changes [30, 43]. For example, multiple ownership on Māori land is difficult to reconcile with current systems and therefore limits access to finance, hinders land use decisions and complicates governance [38]. Similarly, social changes for Māori will be driven by the extent to which land tenure is accompanied by cultural renewal and new social and legal opportunities to decrease inequalities and promote biculturalism, all of which could benefit Māori substantially [40, 44]. However, the process of shifting land tenure risks creating conflicts over land boundaries and management goals and driving disaffection and polarisation in people who feel threatened by the change.

### Native afforestation

Since human arrival, three quarters of indigenous forest cover has been lost nationally, much of which has been converted to commercial pasture and forestry plantations using non-native species [45]. This scenario assumes that commercial forestry and amenity plantings are converted to use exclusively native species and strategic areas like riparian zones and steep slopes are reforested in native plantings for ecosystem services and/or biodiversity credits (permanent forest). This results in significant increases in forest cover with the potential to reverse the decline of native biodiversity, particularly for deliberately planted species. Non-native mammalian herbivore control, restoration of lowland areas and increased connectivity improves resilience of native ecosystems, as does the reduction in propagule pressure for potentially invasive non-native trees [46–48]. However, environmental risks include decreased resilience of tree cover because of transitions and slower tree growth rates and abandonment of non-native production forests. Perverse outcomes could occur if production values conflict with conservation values for native species, for example through a loss of effective genetic diversity for commercially grown native trees. This scenario creates opportunities for policy incentives, such as credits for biodiversity or ecosystem services, strategic tax relief, or practical support to promote managing land for biodiversity and ecosystem services across a broader scale. Such a dramatic transition inherently risks unintended policy consequences and the potential for negative effects on the ability to meet international trade and carbon reduction agreements in the short- to medium-term [49].

The transition away from current non-native forestry practices risks significant economic disruption, with a loss of jobs, drop in export volume and value, and high research and development costs before any new economic return is generated [50]. Credits and other monetary incentives would become an increasingly important component of the national economy, but this risks high exposure to the volatility of such markets. If the transition results in mass job loss and displacement in rural and forestry-dependent communities, this would likely exacerbate inequalities, limit opportunities and increase disaffection. However, if the transition incorporates Māori knowledge and cultural practices, it could improve Māori wellbeing and generate positive social effects from an increased sense of place for all residents [43, 51]. Economic opportunities could develop around creating local and international markets for native plant commodities [52], with the increase in research and development necessary to establish new production forests and markets.

### Predator eradication

Under this scenario, rats (three *Rattus* spp.), mustelids (three *Mustela* spp.) and brushtail possums (*Trichosurus vulpecula*) are functionally suppressed or eliminated nationally [53], and by 2050 funding and operations are shifting to border security and local maintenance. Predator control is expected to increase the population size and range of many native bird species, but should also benefit non-native birds and may result in meso-predator release for uncontrolled non-native predator and herbivore species [54, 55]. Increasing bird populations and a reduction in rodent seed predation will benefit bird-pollinated and bird-dispersed native species, but could also increase invasive plant populations, for example through increased dispersal by birds [56]. Policy opportunities include pioneering management and legislative frameworks to focus on whole-ecosystem regeneration and robust biosecurity and predator eradication. However, later stages of eradication will require major efforts or new technologies to detect and eliminate species at low population densities, potentially leading to controversial or damaging changes to norms and legal rights around private property and unintended harm to non-target species [55]. Significant economic risks stem from the high cost of achieving and maintaining suppression or eradication of target species at large spatial scales. Delayed investment in other sectors as a result of high investment to achieve predator-free status can also result in cascading costs in those neglected sectors [23]. However, once suppression or elimination is achieved, costs of control and costs of intensive intervention for many rare and endangered species are expected to decrease, and new industries and technologies created in the process will provide future opportunities, assuming the sector can pivot once broad-scale predator control is complete.

Many of the cost-benefit arguments made for this scenario assume eradication, however financial benefits may not accrue to the extent expected, particularly if efforts instead achieve suppression [57]. The involvement of community groups in predator control efforts can build engagement, a sense of community and place, and social license for conservation activities [57]. Growing populations and range of native species can also enable the renewal of cultural practices and empower stewardship for indigenous Māori [55]. The extent to which eradication is successful, and the nature of the methods used to achieve or attempt it, will drive many of the social risks, including loss of social license because of high costs, lack of consensus and landowner and Māori consent, unintended consequences, or failure to achieve intended outcomes [55, 58]. Lethal control and the use of poisons can be particularly controversial, both domestically and internationally, and could result in social division and erosion of trust in public and scientific institutions [59, 60].

### Increasing extreme weather events

Extreme weather events increase in frequency, severity and scale, including droughts, fire and storms causing flooding, erosion, windfall and landslides [61]. In addition to point-in-time disruption and destruction, recurring extreme events drive broadly predictable shifts in land use and socio-economic patterns [62]. These changes provide opportunities to prioritize and value ecosystem services and integrity, or to apply new technology to reduce human impacts and improve resilience [63, 64]. Some species will benefit from increasing disturbance, both in their population growth and spread, including native and non-native species [65]. However, increased frequency and severity of mortality events for many species, and fragmentation and disruption of ecosystems, pose the greatest environmental risks, potentially leading to species decline and loss of ecosystem services [21, 66].

Observed increases in weather extremes create policy opportunities by increasing public support and prioritization of climate mitigation, adaptation and resilience planning, which can reduce costs and damage over time [63]. Economic opportunities include new jobs and work arrangements to flexibly adapt to changing conditions, as well as the potential for adaptive policy and management to mitigate financial impacts [67]. However, lags between needs and policy development, excessive demands on funding and resources from multiple, potentially overlapping, crises as well as increasing uncertainty all hinder effective policy development and implementation and risk exacerbating negative impacts [68]. Risks generally outweigh opportunities economically, with high direct costs and subsequent debt, decline in production and productivity, and disruption from forced migration.

Crises can create social opportunities to increase community cohesion [69]. Approaches that emphasize individual and indigenous Māori empowerment can also counter the mental health impacts of these crises and improve decision-making [69, 70]. Increasing extreme events pose serious social risks, however, including increased risk of physical and mental harm, decreased capacity for aid and government agencies to respond, decreased community capacity, and increased inequalities [69, 71]. This may be exacerbated by forced migration, where communities and traditional lands can no longer be inhabited or used in the ways they have been, disrupting social networks and increasing isolation and disaffection [70].

## Discussion

We developed four scenarios, which explore possible futures for 2050 by extending current nascent trends to their full potential expression: increased land ownership and management by indigenous people, afforestation using native trees, predator eradication and increasing extreme weather events. These scenarios are not mutually exclusive and could interact, either amplifying or dampening effects. Although these scenarios were developed for Aotearoa New Zealand, many of the underlying trends are applicable globally, and the overall themes and complications highlighted here are equally relevant in other contexts, particularly as other countries invest in their own ambitious conservation and climate-related projects (e.g. afforestation efforts in treeless environments [72]).

The rights and authority of indigenous people over their ancestral lands is gaining momentum globally, and is part of the growing recognition of crucial linkages between local and indigenous peoples and ecosystems [35, 73, 74]. In Canada, land settlements have been processed through negotiations and tribunals [75], while in the United States Native American reservations have some degree of autonomy but are still heavily influenced by the national-level Bureau of Indian Affairs [76]. In Australia, a much-debated referendum proposing an Aboriginal voice to parliament was recently defeated [77], while indigenous groups in Brazil, though officially recognized as holding land tenure, continue to face loss of land and risk of personal harm as a result of illegal logging and farming [33]. Therefore, a scenario exploring the opportunities and risks associated with the transition of land tenure back to indigenous peoples and increasing integration of indigenous perspectives into national decision-making in conservation can provide meaningful guidance and spark productive conversations in many regions [35, 40].

Similarly, the issue of using non-native vs. native trees for afforestation, forestry and other services has been raised across conservation and carbon capture projects in Africa [78], afforestation efforts in China [79, 80] and more generally with the deliberate afforestation of treeless environments [81]. Knowledge of the risks and benefits of large-scale shifts from non-native to native plants for afforestation highlighted here could help planners avoid many of the potential pitfalls and unintended negative consequences of afforestation efforts.

Non-native predator eradication from increasingly large island systems has been an important conservation tool on remote offshore islands for managing biodiversity over the long-term [82]. Recent eradication efforts on larger inhabited islands, such as South Georgia Island and the Galápagos, highlight the interest in expanding these techniques alongside resident communities, where socio-economic considerations of large-scale management are crucial to include in long-term eradication plans [55].

The urgency of planning for an increase in extreme weather events is already evident globally [18, 19, 83]. Conservation work in the climate change space, however, has often focused on gradual changes driven by altered temperature or rainfall, and our ability to make inferences about extreme events is limited by their lower frequency and higher variability [21]. Nonetheless, extreme events are likely to drive the most severe climate change impacts over the next few decades, both for human society and for conservation. Management and governance often focus on recovery from extreme events, rather than proactively building resilience. Reactive responses are often more costly and risk creating maladaptive outcomes [63, 84].

### Common themes

Several common themes emerged across the four scenarios we developed. Overall, the emerging trends revealed the need for a fundamental change in our approach to valuing and managing the natural environment. National and regional decision-making across sectors needs to place greater weight on maintaining ecological integrity, biodiversity and ecosystem services to mitigate the likely risks and take advantage of the potential opportunities. Many of the most significant implications across scenarios were driven by changes in land use. Land use patterns are well-recognized drivers of biodiversity conservation outcomes, from forest fragmentation to propagule pressure for invasive species [45, 85]. In an economy underpinned by primary production, land use also has significant economic implications, with cascading effects on social fabric and economic opportunities, including conservation funding. Land use patterns can also be sensitive to changes in land ownership, policy, social license and climate. As a result, each of the scenarios we explored reflected anticipated shifts in land use patterns that necessitate a change in land management or goals over the long-term.

The importance of ecosystem services was another theme consistent across the scenarios. How human societies recognize and value ecosystem services has the potential to dramatically affect priorities and decisions around land use and spending [20, 86, 87]. Under the scenario exploring an increase in extreme events, the extent to which ecosystem services form a viable and prioritized part of the national response to climate change was expected to drive many of the conservation outcomes. Similarly, the use of some form of biodiversity or carbon credits was expected to be essential to make a transition from non-native forestry viable in the native afforestation scenario and would likely be an important factor in enabling Māori to achieve their goals under the increased indigenous land tenure scenario.

Each scenario also presented the opportunity to reduce inequality and empower indigenous peoples, but contained the risk that these inequalities could be exacerbated, depending on how policy and management respond to the new challenges. Even under a scenario of increased indigenous land tenure, positive outcomes for Māori communities were not assured, and depended on the way land was transferred and the level of financial support provided during the transition (see also [2, 40]). This emphasizes the importance of governance and policy decisions, which mediate the impact of these scenarios on social structures and can either promote or hinder systemic changes to promote equity and indigenous development across scenarios.

### Next steps and extension to other systems

These scenarios were developed to stimulate future thinking in conservation planning and management. One opportunity provided by a scenarios format is as a tool for engagement across different communities or stakeholders. Scenarios as narratives have the potential to be highly effective for communicating to the public, practitioners, and government and policymakers [15, 17], enabling broader engagement with major emerging environmental issues and the challenges or opportunities they present. In our conversations with land managers and practitioners, the narrative nature of the scenarios has made discussions about the future less abstract and easier to visualize. These discussions have sparked insights and feedback from land managers and practitioners about management challenges under these scenarios, illustrating their use as an engagement tool, providing further guidance to us as we finalized the scenario narratives, and confirming the relevance of the scenarios we chose to develop (R Sprague, per. obs.). Furthermore, scenarios, by making specific hypotheses about the future, also invite constructive critical feedback, through interrogating, adapting and comparing the hypothesized outcomes with real-life events [8]. Discussing these scenarios with experts from other disciplines (e.g. health, technology sectors) can expand the range of implications considered for each scenario, and could draw in other developments, such as disruptive technologies, which are not considered extensively in our narratives. This could also lead to the development of additional scenarios, which reflect the concerns and priorities of fields outside of core conservation ecology disciplines.

Scenarios could be a promising approach to promote future-focused planning for practitioners, policymakers and researchers. For example, the scenarios we developed indicate a need for change in current approaches to resource management decisions to support ecosystem resilience and cultural practices of environmental stewardship. These scenarios could inform critical reviews and reform of relevant policies and legislation, such as the Resource Management Act 1991 and Conservation Act 1987. The involvement of policymakers in our workshop, as well as presentations of the scenarios to policymakers directly, can facilitate translation into policy work, and these scenarios could be included in submissions to local and national governing bodies as they consider new legislation or develop conservation priorities. The scenarios approach explicitly considers the broader socio-economic context in which conservation activities and outcomes occur and the wide range of subjects covered within each scenario benefits practitioners and planners working on environmental and conservation issues from a variety of angles. Scenarios can highlight key decisions or processes that can maximize the opportunities and minimize the risks of expected future trends and reveal uncertainties that would benefit from new knowledge or research. We encourage a long-term outlook that considers decisions and their implications by explicitly considering multi-decadal trends and developments, and that could facilitate more proactive approaches to major emerging issues.
